# Epidemiological Identification of A Novel Pathogen in Real Time: Analysis of the Atypical Pneumonia Outbreak in Wuhan, China, 2019–2020

**DOI:** 10.3390/jcm9030637

**Published:** 2020-02-27

**Authors:** Sung-mok Jung, Ryo Kinoshita, Robin N. Thompson, Natalie M. Linton, Yichi Yang, Andrei R. Akhmetzhanov, Hiroshi Nishiura

**Affiliations:** 1 Graduate School of Medicine, Hokkaido University, Kita 15 Jo Nishi 7 Chome, Kita-ku, Sapporo-shi, Hokkaido 060-8638, Japan; seductmd@med.hokudai.ac.jp (S.-m.J.); kinoshitaryo@gmail.com (R.K.); lukeyang1993@eis.hokudai.ac.jp (Y.Y.); akhmetzhanov@gmail.com (A.R.A.); 2 Mathematical Institute, University of Oxford, Andrew Wiles Building, Radcliffe Observatory Quarter, Woodstock Road, Oxford OX2 6GG, UK; 3 Christ Church, University of Oxford, St Aldates, Oxford OX1 1DP, UK; 4 CREST, Japan Science and Technology Agency, Honcho 4-1-8, Kawaguchi, Saitama 332-0012, Japan

**Keywords:** epidemic, causation, Bayes’ theorem, diagnosis, prediction, statistical model

## Abstract

Virological tests have now shown conclusively that a novel coronavirus is causing the 2019–2020 atypical pneumonia outbreak in Wuhan, China. We demonstrate that non-virological descriptive characteristics could have determined that the outbreak is caused by a novel pathogen in advance of virological testing. Characteristics of the ongoing outbreak were collected in real time from two medical social media sites. These were compared against characteristics of eleven pathogens that have previously caused cases of atypical pneumonia. The probability that the current outbreak is due to “Disease X” (i.e., previously unknown etiology) as opposed to one of the known pathogens was inferred, and this estimate was updated as the outbreak continued. The probability (expressed as a percentage) that Disease X is driving the outbreak was assessed as over 29% on 31 December 2019, one week before virus identification. After some specific pathogens were ruled out by laboratory tests on 5 January 2020, the inferred probability of Disease X was over 49%. We showed quantitatively that the emerging outbreak of atypical pneumonia cases is consistent with causation by a novel pathogen. The proposed approach, which uses only routinely observed non-virological data, can aid ongoing risk assessments in advance of virological test results becoming available.

## 1. Introduction

A cluster of cases of atypical pneumonia with unknown etiology in Wuhan, China attracted global attention towards the end of 2019 [1,2]. An impressive series of rapid virological examinations ruled out common pneumonia-causing viruses such as influenza viruses, adenoviruses, and the coronaviruses associated with Middle East respiratory syndrome (MERS) and severe acute respiratory syndrome (SARS) [2,3,4,5]. Early in the outbreak, the causative agent was suspected to be a coronavirus of non-human origin [5,6]. The coronavirus was subsequently found to be a relative of SARS and named the severe acute respiratory syndrome coronavirus 2 (SARS-CoV-2) [7,8].

While examination of the viral genome was critical for identifying the pathogen, information made publicly available in real time describing clinical characteristics and other outbreak-related factors also allowed experts to consider the etiology and thereby differential diagnoses. For instance, most cases shared a history of visiting or working at a seafood market in Wuhan [3], where exposure to the novel coronavirus is suspected to have occurred with no evidence of direct human-to-human transmission [2], although human-to-human transmission was found later to be common. Observed characteristics of the outbreak led us to believe that the cluster of cases was due to “Disease X” (i.e., an infectious disease of previously unknown viral etiology). However, rigorous quantitative assessment based on these characteristics of the chance that the manifestations of atypical pneumonia were in fact Disease X has not previously been undertaken. The present study addresses this, demonstrating that non-virological information can lead to an objective classification of Disease X, using a simple statistical model that exploits the well-known Bayes’ theorem.

## 2. Methods

### 2.1. Epidemiological Data

As the outbreak unfolded, we calculated in real-time the probability that the pathogen responsible for the atypical pneumonia cases was novel (Disease X), as opposed to the outbreak instead being generated by a previously known pathogen that can cause atypical pneumonia. Our analysis began on 30 December 2019, when the Wuhan Municipal Health Commission announced that there had been a surprisingly large number of atypical pneumonia cases. At that time, we assumed the causative agent could have been one of eight known viral or three known bacterial pathogens, along with the chance that it was instead Disease X. We tracked two active medical social media sites (ProMED [9] and Flutracker [10]) that collected reports of the non-virological characteristics of the outbreak as it progressed. These characteristics were basic observations from the outbreak and do not necessarily represent the features that were causing symptoms. Given these characteristics, we then calculated the probability that the ongoing outbreak was due to a known pathogen or unknown Disease X. On the first day of calculation (i.e., 30 December 2019)—the day that we became aware of the outbreak—the only explanatory factor we included was diagnosis of atypical pneumonia, which was common to all pathogens considered in our dataset. Our analysis represents simple logical deductions from the limited data that were available during the outbreak in a quantitative manner and was updated to reflect new information about the outbreak as it became available in real time.

Table 1 shows the information compiled about the current outbreak, and the dates on which each of these characteristics were discovered. Each characteristic listed was assigned a value of zero or one, denoting whether or not the outbreak characteristic was likely in general (rather than for individual cases) for the emerging outbreak, and the equivalent values for outbreaks of previously observed pathogens were also noted. We note that some information believed at the time was later found to be untrue; for example, it was believed that human-to-human transmission was infrequent. Consequently, inclusion of a large number of characteristics is important for our analysis. Once pathogens were ruled out as the causative agent of the current outbreak, they were removed from our analysis: for example, highly pathogenic avian influenza (HPAI; H5N1) was confirmed not to be the causative agent by laboratory testing on 3 January 2020. Hence, we omitted this pathogen from our analysis from that date onwards. We performed two versions of our analysis to demonstrate how our results might change with the inclusion of different outbreak characteristics. In the first, all characteristics in Table 1 were included in the analysis. In the second, information about the exposure location (i.e., exposure at a wet market) was excluded from the analysis.

### 2.2. Statistical Models

To assess the probability that the emerging outbreak was caused by a known pathogen, we first calculated the distance between the set of characteristics of the ongoing outbreak and those of previously known pathogens. The distance between the characteristics of the ongoing outbreak and cases due to pathogen *j* is denoted by *d_j_*_._ We assumed that the probability that the outbreak is due to a variant of pathogen *j* decreased exponentially with distance *d_j_*. Then, by Bayes’ theorem,

(1) $$\mathrm{Pr}(\mathrm{pathogen}\;j\;|\;\mathrm{observed}\;\mathrm{characteristics})=\frac{\mathrm{Pr}(o\mathrm{bserved}\;\mathrm{characteristics}\;|\;\mathrm{pathogen}\;j)q_{j}}{\sum_{i}\mathrm{Pr}(\mathrm{observed}\;\mathrm{characteristics}\;|\;\mathrm{pathogen}\;i)q_{i}}$$

in which the sum in the denominator is over all possible pathogens *i* (i.e., each of the columns of Table 1, including the column describing the current outbreak). The constants *q_i_* represented a priori probabilities that the outbreak is due to pathogen *i* [11,12]. We set uninformative priors for all pathogens considered, so that *q_i_* was simply the reciprocal of the number of pathogens being considered (including Disease X) on each date in our analysis. We initially estimated the distance between observed characteristics of the outbreak and each known candidate pathogen using the Hamming distance (i.e., the sum of squares differences between the entries in the columns of Table 1 corresponding to the Disease X and the candidate pathogen). Then, we assumed that the probability that the outbreak is driven by pathogen *j* was governed by a negative exponential function,

(2) $$\mathrm{Pr}\left(\mathrm{observed}\;\mathrm{characteristics}\;|\;\mathrm{pathogen}\;j\right)\propto \exp\left(-d_{j}\right)$$

where *d_j_* is the calculated Hamming distance, although in principle any decreasing relationship, and any metric describing the distance between two vectors, could have been used.

We also repeated our analysis using an alternative measure of the distance between observed characteristics of the outbreak and each known candidate pathogen, namely the Euclidean distance (i.e., the square root of the Hamming distance). In each case, we assumed that the importance of each characteristic had an identical weight in our analysis, so that a simple quantitative assessment could be obtained in a probabilistic manner without the need for subjective judgement.

Combining Equations (1) and (2), and assuming uninformative priors for *q_i_*, gives,

(3) $$\mathrm{Pr}(\mathrm{pathogen}\;j\;|\;\mathrm{observed}\;\mathrm{characteristics})=\frac{\exp\left(-d_{j}\right)}{\sum_{i}\exp\left(-d_{i}\right)}$$

The probability that the outbreak was driven by Disease X corresponds to the distance $d_{X}=0$, and represents a risk score taking values between the reciprocal of the number of candidate pathogens including Disease X itself and one:

(4) $$\mathrm{Pr}\left(\mathrm{Disease}\;X\;|\;\mathrm{observed}\;\mathrm{characteristics}\right)=\frac{1}{1+\sum_{i\neq X}\exp\left(-d_{i}\right)}._{\;}$$

If there are *n* known pathogens that can potentially cause atypical pneumonia, the probability of observing Disease X without any information would be identical to the probability of observing any other listed pathogen (i.e., 1/(1 + *n*)). As pathogens were ruled out by laboratory testing, that uninformative probability increased (i.e., 1/12 until 2 January 2020, 1/8 from 3 January 2020 and 1/6 from 5 January 2020 in the current outbreak). In addition, if the probability of observing Disease X according to Equation (3) takes a value close to the probability of observing other candidate pathogens, the overall probability that the outbreak is due to a novel pathogen should be interpreted as being low. A result of significant practical importance, however, is when the probability of observing Disease X is close to one or much larger than the probability corresponding to each previously observed candidate pathogen. In that case, all candidate pathogens are not similar to the causative agent of the ongoing outbreak, and so the outbreak is likely to be due to a novel pathogen.

We converted the probability of Disease X into the equivalent percentage value (so that, for example, a result of 0.8 in Equation (1) is assumed to mean an 80% probability) and refer to the percentage value as the “probability of Disease X” hereafter.

## 3. Results

**Figure 1:**
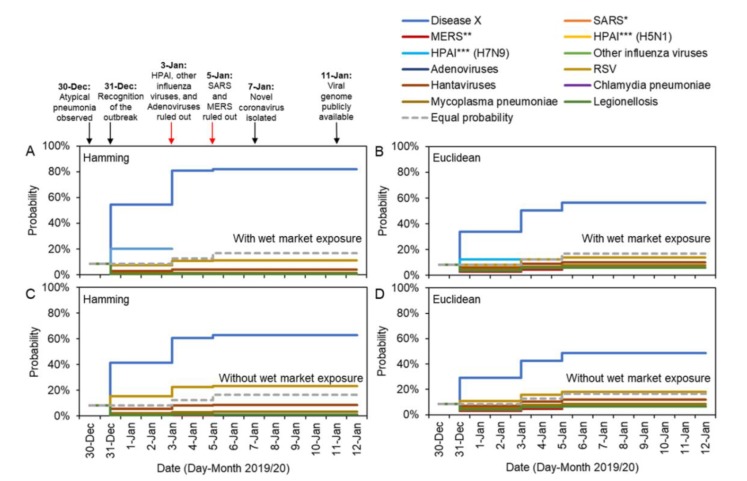
Real-time estimation of the probability that the ongoing pneumonia outbreak is driven by each candidate pathogen, given available information on different days. The probability that the outbreak is due to an unknown pathogen (Disease X) increases as more information becomes available, for two reasons: (i) the current outbreak can be seen to exhibit characteristics that are not similar to those observed in previous outbreaks, and; (ii) previously observed pathogens are ruled out by laboratory test results. Arrows indicate new information available on each date. Results are shown for different metrics describing the distance between characteristics of the ongoing outbreak and each candidate pathogen, and either including or excluding initial exposure information for the current outbreak (i.e., worked at/visited a wet market), specifically: (**A**) Hamming distance (the sum of squares difference between the entries in the columns of Table 1 corresponding to the ongoing outbreak and the candidate pathogen considered) with wet market exposure; (**B**) Euclidean distance (the square root of the Hamming distance) with wet market exposure; (**C**) Hamming distance without wet market exposure; (**D**) Euclidean distance without wet market exposure. Dashed grey lines show the probability for every pathogen (including Disease X) if the only information included is the ruling out of different pathogens through laboratory tests (i.e., a probability of 1/(1 + number of candidate pathogens remaining on that day)). Note that the probability corresponding to different pathogens can be identical, for example, severe acute respiratory syndrome (SARS) and Mycoplasma pneumoniae were assessing as being equally likely as the causative pathogen from 30 December to 4 January, and Legionellosis and Chlamydia pneumoniae had equal probability from 30 December to 12 January (Details in Supplementary Materials Table S1).

We show temporal changes in estimates of the probability that the ongoing outbreak is driven by each candidate pathogen in Figure 1. Because the only information on 30 December 2019 was that cases had symptoms of atypical pneumonia, the distances between the ongoing outbreak and the eleven known pathogens were all zero; thus, all eleven candidate pathogens initially showed an identical probability of 8.3% (i.e., 1/12, when the possibility of Disease X is accounted for). If no further information had become available during the outbreak, other than the gradual ruling out of candidate pathogens through laboratory tests, then the inferred uninformative probability for each candidate pathogen would have been given by the dotted gray lines in Figure 1.

However, additional characteristics of the ongoing outbreak were observed on 31 December 2019. These characteristics allowed the ongoing outbreak to be distinguished from outbreaks due to previous pathogens, and consequently the inferred probability that the outbreak was driven by a novel pathogen increased substantially to 54.3% and 33.8% for Hamming and Euclidean distance metrics, respectively (Figure 1A,B). If instead the exposure characteristic (i.e., exposure at a wet market) was excluded from the analyses, the probability of observing Disease X given observed characteristics was still as high as 41.3% and 29.1% for the Hamming and Euclidean distance metrics (Figure 1C,D).

Adenoviruses, HPAI (H5N1 and H7N9) and other influenza viruses were ruled out on 3 January 2020, leading to an estimated probability that the outbreak was due to Disease X of 80.8% and 50.2% for the Hamming and Euclidean distance metrics when all factors were considered. Excluding the characteristic corresponding to wet market exposure, the probability that the outbreak was due to Disease X was assessed to be 60.7% and 42.7% for the Hamming and Euclidean distance metrics, respectively. SARS and MERS coronaviruses were ruled out as possible causative agents on 5 January 2020, leading to a very high estimate for the probability that the outbreak was caused by a novel pathogen once all information was collected. On 12 January 2020, the probability the outbreak was due to Disease X was estimated to be 82.2% and 56.5% according to the model considering all the characteristics (again, for the Hamming and Euclidean distances, respectively), while the model excluding the characteristic of exposure at the wet market suggested probabilities of 62.9% and 48.6%.

## 4. Discussion

In this analysis, we showed how the outbreak of pneumonia cases in Wuhan was assessed in early January 2020 as being caused by a novel pathogen. This was demonstrated using a series of clinical, occupational, and behavioral observations extracted from fragmented reports describing the cases as these reports became available in real time [3,6]. Although virological investigation is the gold standard for pathogen identification, and the virus has now been confirmed to be a novel coronavirus that is a relative of SARS, laboratory-based outcomes can only be obtained after successfully sequencing the novel virus, which can sometimes be a lengthy process. At the time of writing, it still remains for the microbiological causal link to be established, for instance by ensuring that Koch’s postulates are met (as seen, e.g., in a study of Zika virus [13]). In the ongoing outbreak, the provisional identification of a novel coronavirus was performed on 7 January 2020 and announced formally on 9 January 2020 [2]. We have shown that non-virological information can indicate that the cause of the outbreak is likely to be a novel pathogen (“Disease X”), and that this conclusion was obtained before virological test results were announced. Disease X was inferred to be very likely on all dates from 31 December 2019 onwards—the date on which descriptions of outbreak characteristics began to emerge.

When sufficient clinical details of cases (e.g., complete blood cell counts) are available, the number of causative pathogens considered can be limited to a reasonable number. In this instance, atypical pneumonia combined with reduced white blood cell counts and the lack of response to antibiotics indicated that the pathogen was consistent with viral rather than bacterial infection. With such information, non-virological data can be used for convenient quantification of the probability that the outbreak was due to a novel pathogen, while awaiting the results of virological tests. We believe that the proposed approach can improve risk assessment practices across the world.

It is important to consider two issues about the compilation of Table 1. First, a critical underlying assumption is that Table 1 represents general outbreak characteristics of the ongoing outbreak and previously known outbreaks. The representation does not reflect observations from all confirmed cases nor epidemiological findings from a case control study (e.g., statistically significant risk factors). Rather, zeros and ones in the table were defined in a phenomenological manner, and values may change as the ongoing outbreak continues. Depending on the opinions of different experts (e.g., [14]), the defined nominal values could have been different to those shown in Table 1; in this study, we are simply demonstrating how such an approach might work in practice. Second, as we have shown, quantitative estimates depend on the precise characteristics that are used. We showed results including and not including information on wet market exposure. In Table 1, infections due to previously observed pathogens other than HPAI were assumed not to be associated with exposure to wet markets. Since this assumption was not derived from empirical observations, it could be debated.

In the past, descriptive outbreak information has been used to generate outbreak case definitions, and causative agents have been pinpointed without using statistical methods in combination with epidemiological observations. In the present study, we have shown that such assessments can be made quantitatively using a simple statistical model, allowing for comparisons between the possible causative agents among different candidates. When outbreak characteristics are shared and updated in real-time (Table 1), these data can contribute to efforts to narrow down the possible range of causative agents. In the case of the outbreak in Wuhan, our calculation of the probability that each pathogen is the causative agent indicates that virological exclusion of influenza viruses, adenoviruses and known virulent coronaviruses associated with SARS and MERS on 3 and 5 January 2020 can be regarded as an “unsurprising” finding.

As important limitations, the precision and credibility of the input data and the method for calculating the distance between the candidate pathogens and the observed outbreak, must be refined in future. First, our proposed approach used very limited data in Table 1 for logical quantification of the probability that each pathogen was the causative agent. However, with more clinical data, the binary characteristics could be replaced by continuous frequencies (e.g., the proportion of cases experiencing coughing and/or breathing difficulties). Second, with sufficient data it would also be possible to estimate the probability that each pathogen is the causal agent (Equation (1)) not by arbitrarily measuring the distance but by using classification models involving regression or more sophisticated machine learning approaches. Third, the erroneous input of incorrect information may be a challenge in real time analyses. The veracity of the sources of information for future analyses could have an impact on the resulting probability calculations. Fourth, the estimated probability that an outbreak is driven by a novel pathogen might be slightly over- or underestimated due to limited information about the mode of transmission and small numbers of observed cases. Of note, the respiratory syncytial virus (RSV) was not completely ruled out as a candidate pathogen in our real-time analysis. However, RSV was an unlikely candidate since the majority of cases in the ongoing outbreak are adults [15] while most RSV infections are observed in infants and young children. Finally, we had to restrict ourselves to assuming the a priori probability that the ongoing outbreak driven by each candidate pathogens ($q_{i}$) is identical for each pathogen. However, since no alternative information was available, we believe such uninformative priors to be the optimal choice.

Despite the future improvements to our statistical modelling framework that are required, including the need to test our approach using data from outbreaks of previously known pathogens, this short study demonstrated clearly that the ongoing outbreak is consistent with causation by a novel pathogen, “Disease X”. We reached this conclusion after only a few days of the outbreak had passed. Attention has now rightly turned towards identifying the pandemic potential of this outbreak [16,17,18], as well as planning control interventions within China and elsewhere [19,20]. However, at the start of the next outbreak of an unknown pathogen, virological testing and quantitative analyses of clinical data are two complementary methods that can be used. Thus, analyses of the type conducted in this study can greatly support efforts to characterize causal agents in future outbreaks, with the benefit that analyses like this one can be carried out extremely quickly.

## Figures and Tables

**Table 1:** Observed characteristics of the current outbreak in Wuhan, China, as well as general characteristics of outbreaks driven by previously known pneumonia-causing pathogens.

Category	Characteristic	Current Outbreak		Viral Outbreaks								Bacterial Outbreaks		
		Disease X	Date Info Shared	SARS	MERS	HPAI (H5N1)	HPAI (H7N9)	Other Influenza Viruses	Adenoviruses	Hantaviruses	RSV	*Chlamydia pneumoniae*	*Mycoplasma pneumoniae*	Legionellosis
Clinical	Atypical pneumonia	1	30-Dec	1	1	1	1	1	1	1	1	1	1	1
Clinical	CT (pulmonary infiltrates)	1	31-Dec	1	1	1	1	0	0	1	1	1	1	1
Clinical	Low white blood cell counts	1	31-Dec	1	1	1	1	1	1	1	1	0	0	0
Clinical	No response to antibiotics	1	31-Dec	1	1	1	1	1	1	1	1	0	0	0
Clinical	Frequent human-to-human transmission	0	31-Dec	1	1	0	0	1	1	0	1	1	1	1
Clinical	Substantial lethal cases	0	31-Dec	1	1	1	1	0	0	1	0	0	0	0
Travel/Occupation	Worked at/visited a wet market	1	31-Dec	0	0	1	1	0	0	0	0	0	0	0
Travel/Occupation	Worked at/visited a hospital	0	31-Dec	1	1	0	0	0	0	0	0	0	0	0
Travel/Occupation	Visited Middle Eastern countries	0	31-Dec	0	1	0	0	0	0	0	0	0	0	0
Travel/Occupation	Visited hot spring or contact with potable water	0	31-Dec	0	0	0	0	0	0	0	0	0	0	1
Zoonotic	Contact with camels	0	31-Dec	0	1	0	0	0	0	0	0	0	0	0
Zoonotic	Contact with parrots/wild birds	0	31-Dec	0	0	1	0	0	0	0	0	1	0	0
Zoonotic	Contact with rodents	0	31-Dec	0	0	0	0	0	0	1	0	0	0	0

SARS, Severe acute respiratory syndrome; MERS, Middle East respiratory syndrome; HPAI, Highly pathogenic avian influenza; RSV, Respiratory syncytial virus. Zeros represent characteristics that are unlikely for outbreaks of that pathogen, and ones represent characteristics that occur. Dates and characteristics for the ongoing outbreak were obtained from two online information systems [7,8], and information for other pathogens was summarized from the pathogen-specific pages on the WHO and CDC websites.

## Supplementary Materials

The following are available online at https://www.mdpi.com/2077-0383/9/3/637/s1, Table S1: Estimated values of the probability of Disease X, given available information at different timepoints using Hamming distance and including wet market exposure, Table S2: Estimated values of the probability of Disease X, given available information at different timepoints using Euclidean distance and including wet market exposure, Table S3: Estimated values of the probability of Disease X, given available information at different timepoints using Hamming distance and excluding wet market exposure, Table S4: Estimated values of the probability of Disease X, given available information at different timepoints using Euclidean distance and excluding wet market exposure.
