# Supplementary material for: Epidemiological Identification of A Novel Pathogen in Real Time: Analysis of the Atypical Pneumonia Outbreak in Wuhan, China, 2019–2020
Source: J Clin Med. 2020 Feb 27;9(3):637. doi: 10.3390/jcm9030637 (PMC7141128; doi:10.3390/jcm9030637)
Supplement: Supplementary file 1 [file jcm-09-00637-s001.pdf]

Table S1. Estimated values of the probability of Disease X, given available information at different timepoints using Hamming distance and including wet market exposure.

Hamming distance, with wet market													
Date	Disease X	SARS	MERS	HPAI (H5N1)	HPAI (H7N9)	Other Influenza viruses	Adenoviruses	Hantaviruses	RSV	Chlamydia pneumoniae	Mycoplasma pneumoniae	Legionellosis	Equal probability
30-Dec	8.3%	8.3%	8.3%	8.3%	8.3%	8.3%	8.3%	8.3%	8.3%	8.3%	8.3%	8.3%	8.3%
31-Dec	54.3%	1.0%	0.1%	7.4%	20.0%	2.7%	2.7%	2.7%	7.4%	0.4%	1.0%	0.4%	8.3%
1-Jan	54.3%	1.0%	0.1%	7.4%	20.0%	2.7%	2.7%	2.7%	7.4%	0.4%	1.0%	0.4%	8.3%
2-Jan	54.3%	1.0%	0.1%	7.4%	20.0%	2.7%	2.7%	2.7%	7.4%	0.4%	1.0%	0.4%	8.3%
3-Jan	80.8%	1.5%	0.2%					4.0%	10.9%	0.5%	1.5%	0.5%	12.5%
4-Jan	80.8%	1.5%	0.2%					4.0%	10.9%	0.5%	1.5%	0.5%	12.5%
5-Jan	82.2%							4.1%	11.1%	0.6%	1.5%	0.6%	16.7%
6-Jan	82.2%							4.1%	11.1%	0.6%	1.5%	0.6%	16.7%
7-Jan	82.2%							4.1%	11.1%	0.6%	1.5%	0.6%	16.7%
8-Jan	82.2%							4.1%	11.1%	0.6%	1.5%	0.6%	16.7%
9-Jan	82.2%							4.1%	11.1%	0.6%	1.5%	0.6%	16.7%
10-Jan	82.2%							4.1%	11.1%	0.6%	1.5%	0.6%	16.7%
11-Jan	82.2%							4.1%	11.1%	0.6%	1.5%	0.6%	16.7%
12-Jan	82.2%							4.1%	11.1%	0.6%	1.5%	0.6%	16.7%

SARS, Severe acute respiratory syndrome; MERS, Middle East respiratory syndrome; HPAI, Highly pathogenic avian influenza; RSV, Respiratory syncytial virus.

Table S2. Estimated values of the probability of Disease X, given available information at different timepoints using Euclidean distance and including wet market exposure.

Euclidean distance, with wet market

Date	Disease X	SARS	MERS	HPAI (H5N1)	HPAI (H7N9)	Other Influenza viruses	Adenoviruses	Hantaviruses	RSV	Chlamydia pneumoniae	Mycoplasma pneumoniae	Legionellosis	Equal probability
30-Dec	8.3%	8.3%	8.3%	8.3%	8.3%	8.3%	8.3%	8.3%	8.3%	8.3%	8.3%	8.3%	8.3%
31-Dec	33.8%	4.6%	2.9%	8.2%	12.4%	6.0%	6.0%	6.0%	8.2%	3.6%	4.6%	3.6%	8.3%
1-Jan	33.8%	4.6%	2.9%	8.2%	12.4%	6.0%	6.0%	6.0%	8.2%	3.6%	4.6%	3.6%	8.3%
2-Jan	33.8%	4.6%	2.9%	8.2%	12.4%	6.0%	6.0%	6.0%	8.2%	3.6%	4.6%	3.6%	8.3%
3-Jan	50.2%	6.8%	4.3%					8.9%	12.2%	5.4%	6.8%	5.4%	12.5%
4-Jan	50.2%	6.8%	4.3%					8.9%	12.2%	5.4%	6.8%	5.4%	12.5%
5-Jan	56.5%							10.0%	13.7%	6.0%	7.6%	6.0%	16.7%
6-Jan	56.5%							10.0%	13.7%	6.0%	7.6%	6.0%	16.7%
7-Jan	56.5%							10.0%	13.7%	6.0%	7.6%	6.0%	16.7%
8-Jan	56.5%							10.0%	13.7%	6.0%	7.6%	6.0%	16.7%
9-Jan	56.5%							10.0%	13.7%	6.0%	7.6%	6.0%	16.7%
10-Jan	56.5%							10.0%	13.7%	6.0%	7.6%	6.0%	16.7%
11-Jan	56.5%							10.0%	13.7%	6.0%	7.6%	6.0%	16.7%
12-Jan	56.5%							10.0%	13.7%	6.0%	7.6%	6.0%	16.7%

SARS, Severe acute respiratory syndrome; MERS, Middle East respiratory syndrome; HPAI, Highly pathogenic avian influenza; RSV, Respiratory syncytial virus.

Table S3. Estimated values of the probability of Disease X, given available information at different timepoints using Hamming distance and excluding wet market exposure.

Hamming distance, without wet market													
Date	Disease X	SARS	MERS	HPAI (H5N1)	HPAI (H7N9)	Other Influenza viruses	Adenoviruses	Hantaviruses	RSV	Chlamydia pneumoniae	Mycoplasma pneumoniae	Legionellosis	Equal probability
30-Dec	8.3%	8.3%	8.3%	8.3%	8.3%	8.3%	8.3%	8.3%	8.3%	8.3%	8.3%	8.3%	8.3%
31-Dec	41.3%	2.1%	0.3%	5.6%	15.2%	5.6%	5.6%	5.6%	15.2%	0.8%	2.1%	0.8%	8.3%
1-Jan	41.3%	2.1%	0.3%	5.6%	15.2%	5.6%	5.6%	5.6%	15.2%	0.8%	2.1%	0.8%	8.3%
2-Jan	41.3%	2.1%	0.3%	5.6%	15.2%	5.6%	5.6%	5.6%	15.2%	0.8%	2.1%	0.8%	8.3%
3-Jan	60.7%	3.0%	0.4%					8.2%	22.3%	1.1%	3.0%	1.1%	12.5%
4-Jan	60.7%	3.0%	0.4%					8.2%	22.3%	1.1%	3.0%	1.1%	12.5%
5-Jan	62.9%							8.5%	23.1%	1.2%	3.1%	1.2%	16.7%
6-Jan	62.9%							8.5%	23.1%	1.2%	3.1%	1.2%	16.7%
7-Jan	62.9%							8.5%	23.1%	1.2%	3.1%	1.2%	16.7%
8-Jan	62.9%							8.5%	23.1%	1.2%	3.1%	1.2%	16.7%
9-Jan	62.9%							8.5%	23.1%	1.2%	3.1%	1.2%	16.7%
10-Jan	62.9%							8.5%	23.1%	1.2%	3.1%	1.2%	16.7%
11-Jan	62.9%							8.5%	23.1%	1.2%	3.1%	1.2%	16.7%
12-Jan	62.9%							8.5%	23.1%	1.2%	3.1%	1.2%	16.7%

SARS, Severe acute respiratory syndrome; MERS, Middle East respiratory syndrome; HPAI, Highly pathogenic avian influenza; RSV, Respiratory syncytial virus.

Table S4. Estimated values of the probability of Disease X, given available information at different timepoints using Euclidean distance and excluding wet market exposure.

Euclidean distance, without wet market

Date	Disease X	SARS	MERS	HPAI (H5N1)	HPAI (H7N9)	Other Influenza viruses	Adenoviruses	Hantaviruses	RSV	Chlamydia pneumoniae	Mycoplasma pneumoniae	Legionellosis	Equal probability
30-Dec	8.3%	8.3%	8.3%	8.3%	8.3%	8.3%	8.3%	8.3%	8.3%	8.3%	8.3%	8.3%	8.3%
31-Dec	29.1%	5.1%	3.1%	7.1%	10.7%	7.1%	7.1%	7.1%	10.7%	3.9%	5.1%	3.9%	8.3%
1-Jan	29.1%	5.1%	3.1%	7.1%	10.7%	7.1%	7.1%	7.1%	10.7%	3.9%	5.1%	3.9%	8.3%
2-Jan	29.1%	5.1%	3.1%	7.1%	10.7%	7.1%	7.1%	7.1%	10.7%	3.9%	5.1%	3.9%	8.3%
3-Jan	42.7%	7.6%	4.6%					10.4%	15.7%	5.8%	7.6%	5.8%	12.5%
4-Jan	42.7%	7.6%	4.6%					10.4%	15.7%	5.8%	7.6%	5.8%	12.5%
5-Jan	48.6%							11.8%	17.9%	6.6%	8.6%	6.6%	16.7%
6-Jan	48.6%							11.8%	17.9%	6.6%	8.6%	6.6%	16.7%
7-Jan	48.6%							11.8%	17.9%	6.6%	8.6%	6.6%	16.7%
8-Jan	48.6%							11.8%	17.9%	6.6%	8.6%	6.6%	16.7%
9-Jan	48.6%							11.8%	17.9%	6.6%	8.6%	6.6%	16.7%
10-Jan	48.6%							11.8%	17.9%	6.6%	8.6%	6.6%	16.7%
11-Jan	48.6%							11.8%	17.9%	6.6%	8.6%	6.6%	16.7%
12-Jan	48.6%							11.8%	17.9%	6.6%	8.6%	6.6%	16.7%

SARS, Severe acute respiratory syndrome; MERS, Middle East respiratory syndrome; HPAI, Highly pathogenic avian influenza; RSV, Respiratory syncytial virus.
